# Influencing factors of delay in seeking medical attention of patients with obstructive sleep apnea based on the Model of Pathways to Treatment in China: a qualitative analysis

**DOI:** 10.1007/s11325-024-03078-1

**Published:** 2024-06-18

**Authors:** Hongyan Shang, Dandan Chen, Qingmei Deng, Zuchang Ma

**Affiliations:** 1 Department of Thoracic Oncology, Hefei Cancer Hospital, Chinese Academy of Sciences, Hefei, 230088 Anhui Province China; 2 Department of Nursing, Hefei Cancer Hospital, Chinese Academy of Sciences, Hefei, 230088 Anhui Province China; 3 Department of Laboratory, Hefei Cancer Hospital, Chinese Academy of Sciences, Hefei, 230088 Anhui Province China; 4 Hefei Institutes of Physical Science, Chinese Academy of Sciences, Hefei, 230031 Anhui Province China

**Keywords:** Treatment delay, Seeking medical attention, Obstructive sleep apnea, The Model of Pathways to Treatment

## Abstract

**Background:**

There is the highest estimated number of patients with obstructive sleep apnea (OSA) in China. Early treatment could lead to fewer complications associated with OSA. This study aimed to analyze the factors influencing help-seeking from the first symptom discovery to treatment in OSA.

**Methods:**

Semi-structured interview outline was designed to conduct face-to-face interview based on the analyses of a great number of related literatures on the delay in seeking medical attention of patients with OSA. 15 patients diagnosed were interviewed between June 2021 to September 2022 in general hospital of Shenyang, Northeastern of China. Qualitative data was analyzed by content analysis using the Model of Pathways to Treatment.

**Results:**

Analyses identified factors contributing to elapsed time from first symptom discovery to received treatment that are linked to disease characteristic, patients, health system organization. Appraisal interval is most obvious for patients with OSA, but it is difficult to pinpoint precisely because the patients didn’t remember exactly when the first symptom was detected.

**Conclusions:**

Patients diagnosed with OSA didn’t initially interpret the snore as a warning sign and even thought it was a blessing. The findings provided guidance or avenues for reducing elapsed time between the first symptom and received treatment.

## Introduction

Obstructive sleep apnea (OSA) is a common sleep disorder caused by repeated upper airway partial or total collapse at night during sleep, resulting in hypopnea and a drop in blood oxygen [1]. It could bring about different degrees of chronic intermittent hypoxia and hypercarbia, often accompanied by daytime sleepiness, fatigue, depression and other symptoms, which further lead to the other multi-system lesions and damage including cardiovascular system, endocrine system, nervous system and so on. Evidence suggests that the incidence rates of OSA has showed a gradual increasing trend [2].

However, the medical treatment status of OSA patients is not optimistic. Furthermore, it is a very important thought-provoking problem that delay in medical treatment of OSA patients. Finkel et al. [3, 4] pointed out that 82% of men and 93% of women in patients with moderate and severe OSA hadn’t been diagnosed definitively due to that they were not aware of their snoring symptom at night and did not seek medical attention in time. Henry [5] conducted semi-structured interviews with 24 OSA patients and their families, and pointed out that the average delay in seeking medical treatment for OSA patients was (4.8 ± 5.8) years, (5.5 ± 8.7) years for male patients and (4.0 ± 3.4) years for female patients. It was reported that the fatality rate was 13% of OSA patients who were not diagnosed and treated timely due to delayed medical treatment within 5 years, and 37% of OSA patients with apnea hypopnea index (AHI) above 20 within 8 years [6]. What is the status quo of the delays of 176 million OSA patients in China? And what are the influencing factors for them?

The objective of the article is to identify the influencing factors delay in seeking medical attention of patients with OSA. It is necessary to understand the process of delayed medical treatment in OSA patients and its related influencing factors for medical personnel and healthcare policymakers. However, there were few studies on the delay of medical treatment of OSA at home and abroad, which didn’t pay attention to the delay of diagnosis and treatment, and lack of theoretical support. The study attempted to apply the Model of Pathways to Treatment originally applied to cancer research to the study of delay in seeking medical attention of OSA.

### New contribution

The contribution of the article is threefold. First, the Model of Pathways to Treatment was applied in patients with OSA. According to the theory, the results reveal that the delay in appraisal interval is most obvious to patients with OSA, which provide a certain direction for the formulation of relevant intervention policies. More potential influencing factors that may delay medical treatment could be uncovered by semi-open-ended questions, which can be divided into individual, the health service system and the disease. Moreover, the results show that to improve delay in seeking medical treatment requires the efforts of individuals, society and the state.

## Methods

### Conceptual framework

**Fig. 1:**
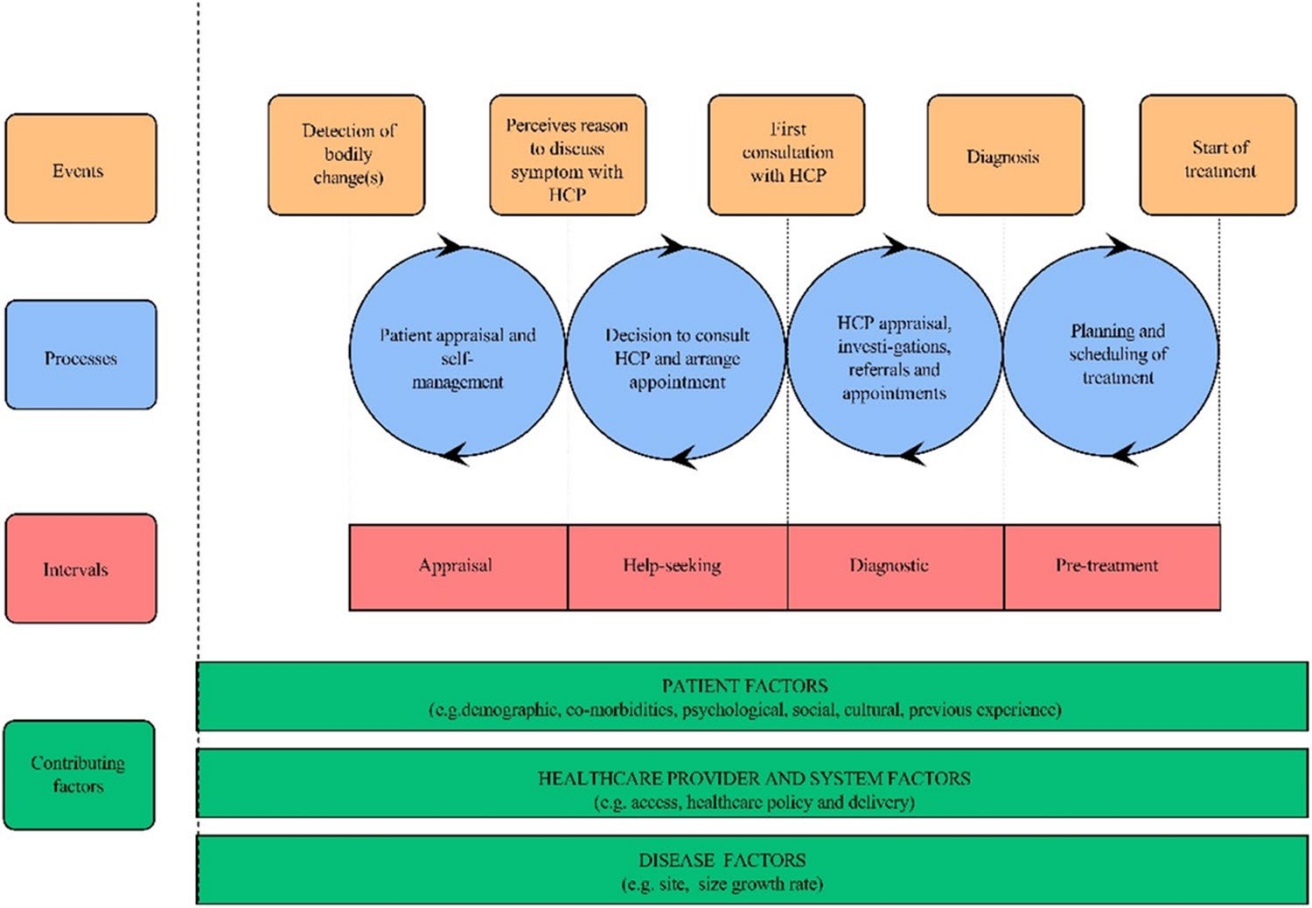
The Model of pathways to treatment [8] (HCP, healthcare professional)

The Model of Pathways to Treatment was originated and refined from the Andersen’s Model of Total Patient Delay [7]. There were five stages: appraisal delay, illness delay, behavioral delay, scheduling delay, treatment delay in Andersen’s Model of Total Patient Delay. However, it has the flaws of lack of specification of time intervals measured for illness delay and appraisal delay. And it was difficult to confirm the exact duration of the behavior delay. Thus, the Model of Pathways to Treatment was modified by Walter [8] (see Fig. 1). The refinement of Andersen’s model identified four intervals: appraisal interval, help-seeking interval, diagnostic interval, pre-treatment interval. The appraisal interval refers to the period of time between the onset of somatic symptoms and the awareness to seek medical attention. The help-seeking interval is the period a person conscious of seeking medical attention decides to consult up to the first visit with a physician. The diagnostic interval is the interval between a person consulting up to the first visit with a physician and be diagnosed definitely after various tests and examinations. The pre-treatment interval could be defined as the hesitating stage before a planned treatment. There are serious advantages of The Model of Pathways to Treatment. The first and most important feature is that the starting point of each stage can be identified by patients, clinicians and researchers distinctly. Secondly, many influencing aspects have been clearly identified during the whole process of delays in seeking medical care by The Model of Pathways to Treatment, such as the individual, the health service system and the disease. The Model of Pathways to Treatment had been applied ever since it was proposed in many studies, which is widely used in the research of tumors, such as colorectal cancer, female tumors with high incidence, brain tumors. It is well suited to elaborate entire process of delays in seeking medical attention of OSA with The Model of Pathways to Treatment for that the theory has a good predictive and explanatory power on medical seeking behavior.

### Study design and collection and analysis of qualitative data

The qualitative research presented here was conducted among 15 adult patients with OSA to reveal the factors influencing the delays in seeking medical attention between first symptom and treatment with adult patients with OSA. The enrolled subjects were located in three provinces in northeast China.

We defined the operational definition of delay in seeking medical attention with adult patients with OSA on the basis of following these steps. Delay in seeking medical attention was defined as the time interval between the onset of moderate to severe snoring symptoms and the first visit to the doctor for the symptom of OSA being more than 3 months. First, we have carried through literature reviews for the purpose of forming operational definition of delaying in seeking medical attention with adult patients with OSA. According to the selection criteria, 5 experts in related fields were selected and invited. The selection criteria for experts were as follows: (a) Higher academic level in OSA treatment and nursing; (b) Rich experience in clinical treatment and nursing; (c) Be familiar with the development of measuring tools and measuring methods of psychometric characteristics; (d) Associate senior or above professional titles. Then we had an expert consultation by Delphi method for an uncertainty. The uncertainty stems from the fact that OSA is essentially a different disease from cancer. As for cancer, the interval time that three months or over was defined as delay in seeking medical attention. What about for OSA? Or that, the interval time that between the onset of somatic symptoms and serious complication of OSA was defined as delay in seeking medical attention. Which one was more appropriate for OSA? Finally, the perspectives or recommendations of experts were asked in person or by email about the definition of delay in seeking medical attention. Two rounds of expert consultations had been conducted until expert consensus is reached.

The project has been approved by the Ethics Committee of the First Affiliated Hospital of China Medical University. Before the start of the study, the purpose and significance of the study were explained to the patients and their families, and then the informed consent was signed with the participant to ensure that the investigation results were only used in the study and the strict confidentiality principle should be followed.

Semi-structured interviews lasting approximately 30 min to one hour were conducted by the researcher and were audio-recorded with the respondents’ consent. According to the willingness of the participants, the interview was conducted in the quiet environment, such as a sleep clinic laboratory, a group meeting room or the participants’ home. All interviews were transcribed and subsequently analyzed by the method of theme analysis within 24 h. The facts that the initial symptom of respondents’, the reasons for not seeking medical attention in time, and the barriers encountered during the medical treatment, such as financial difficulties and complicated medical procedures were explored by open-ended questions. When patients forgot the specific date of their first symptom, they would been given some questions that the events of special significance of the life designed to minimize memory bias, such as “were you married at the time? did you already have your first baby? and how old was the first baby?” We conducted 15 in-depth interviews between June 2021 and September 2022. All interviews were double-coded separately by two researchers. If there were any divergences, it would be discussed in the team meetings in order to reach a consensus. We have summarized and analyzed each subject’s treatment pathway. When there were no more new themes, that was, theme saturation, the interview was stopped. However, we can’t say we’ve explored all the examples because of the diversity of the stories.

## Results

The characteristics of 15 patients with OSA in the interview were displayed in Table 1. A large proportion of onset symptoms of OSA could be easily recognized except that a fraction of symptoms is less typical, such as encephalalgia, fatigue. For example, respondent 13 finally was recommended for polysomnography (PSG) after seeking medical recommendations of a lot of different departments for her atypical headache. We have arranged the appraisal interval from six months to 50 years (see Table 2).

**Table 1:** The characteristics of 15 patients with obstructive sleep apnea (*N* = 15)

#	Gender	Age	BMI	Marital status	Educational level	Family history	Neck circumference (cm)	Maximum apnea duration(s)	AHI	LSpO_2_ (%)	Clinical symptom	Complication(s)
1	Male	28	29.1	Single	Postgraduate	-	41.0	39.0	58.6	84.0	Fatigue	High blood pressure
2	Male	47	31.7	Married	Junior college	+	43.0	63.5	60.2	60.0	Apnea	No complication
3	Male	56	27.7	Married	Senior Secondary School	+	40.0	42.0	44.7	78.0	Polyp of vocal cord	High blood pressure
4	Male	40	28.5	Married	Undergraduate	+	39.0	62.0	29.6	76.0	No clinical symptom	Gastroesophageal reflux and heart trouble
5	Male	57	29.7	Married	Illiteracy	+	42.0	26.0	52.8	75.0	Apnea	No complication
6	Male	66	24.5	Married	Undergraduate	+	39.0	55.0	23.3	81.0	Apnea	No complication
7	Female	69	25.2	Married	Illiteracy	+	40.0	31.0	46.4	87.0	No clinical symptom	Heart trouble
8	Male	34	26.4	Married	Graduate	-	42.0	39.8	17.7	87.0	Apnea	No complication
9	Male	42	33.0	Married	Undergraduate	+	44.0	60.0	59.9	61.0	No clinical symptom	High blood pressure and heart trouble
10	Male	49	28.9	Married	Graduate	+	41.0	38.0	13.9	88.0	Hypertrophy of tonsil	No complication
11	Female	40	26.2	Married	Junior high school degree	+	42.0	80.0	27.5	75.0	Apnea	Heart trouble
12	Male	55	25.1	Married	Undergraduate	+	41.0	50.5	46.5	82.0	Fatigue	No complication
13	Female	54	25.4	Married	Vocational high schools	+	40.0	63.0	59.4	75.0	Headache	No complication
14	Female	56	23.9	Married	Junior college	+	40.0	58.5	77.7	67.0	No clinical symptom	High blood pressure
15	Female	40	39.8	Single	Technical secondary school	+	49.0	61.5	58.4	82.0	No clinical symptom	High blood pressure

**Table 2:** Patient pathways of obstructive sleep apnea (*N* = 15)

#	Appraisal interval	Help-seeking interval	Diagnostic interval	From first symptom to diagnosis
4	6 months	2 months	2 weeks	I did not take it seriously, heart pain, something in my stomach seems to be upturning. I’ve taken an examination in the local hospital (Panjin). The doctor advised me to hang a bag (24 h dynamic electrocardiogram), and then he (the doctor) said premature beat. He said this (PSG), (said) if sleep was cured, the heart would be healed. I sought the doctor for the first time in Panjin Hospital on September 20th. I knew snoring was a kind of disease before, but I didn’t take it seriously. My family also knew it was a disease, and they didn’t think it was so serious, (PSG) not easy to registration, our city hospital (diagnosis and treatment technology) was poor, (doctor) directly advised me to come to here, or go to Beijing
8	2 years	1 year	2 months	I’ve been snoring ever since my son was born. This time is because of choking breath. I can’t sleep on my back and feel it aggravated recently. My comrade was dead in this sleep last year. He has a larger neck circumference. I laid down and couldn’t breathe, all this exactly same thing with him, so I went to ENT first. Before (I knew) snoring was a kind of disease, but did not expect so serious and heard that Gao Cumin (a celebrity) was died of the disease. Now I would have been suffocated to wake up in some minutes after fall sleep, and had a heavy-headedness, dry mouth. In order to find this disease, I’ve been to a lot of outpatient departments. First went to ENT, and then go to the respiratory department doctor
1	5 years	2 months	1 week	I knew that snoring is a medical condition. The doctor suggested me to have a PSG, because I have a high blood pressure, suggested that I did this. And I was aware of snoring for a long time. I’m a veteran. I used to live in the army dormitory, and found that we all snored. Almost all the men snored, so you can’t say they were all sick. Only the ones that snored really loud to be considered sick. The examination is troublesome. My parents are older, my wife broke a bone, and I have a baby, so I have to make an appointment in advance. I’m lucky. (Because) I had an appointment in the morning and was told there was an available bed for PSG in the afternoon. Sometimes there are too many people to make an appointment, right? The trouble is, I have to find someone to accompany me
3	5 years	2 years	3 days	I went to Liaoning Hospital of Traditional Chinese Medicine the day before yesterday because of polyps of vocal cords, and the doctor said: “Your vocal cords don’t seem to be very terrible, maybe it’s snoring. Your snoring is very serious, and you should pay more attention to it.” He said that the high blood pressure was related to snoring. After running and no smoking for two years, blood pressure (still high), has not gone down. And then I felt breathing trouble, the doctor said to have the laryngoscope and let me do this (PSG). I’m going to have an operation soon to cut the vocal cords. I think it can do two things with one dollar. Why not do it? I used to know that I was snoring, but I didn’t pay attention to it. It didn’t affect my life
9	10 years	6 months	2 days	I didn’t know it was a disease. There’s an old saying that snoring is a blessing. My friend said that this (snoring) is a kind of disease and suggested me to a medical college. At that time (I) also did not take it seriously. Later, I was diagnosed as high blood pressure. He (the friend) said all this had to do with snoring. I registered the cardiology department and had a test yesterday, also wore a dynamic heart rate box (24 h dynamic electrocardiogram) all day. Then irregular heart rate. (Doctor) said this (snoring) may have an impact, so I went to the respiratory department and had this monitoring (PSG). I don’t think it’s any trouble to do this examination. Compared with other examinations, this examination is very simple, just come to get a 7-h sleep
15	10 years	1 year	3 weeks	I didn’t know if I was snoring and didn’t really care. Actually, I used to snore for many years. I wondered if it was normal for snoring, so I had made an appointment with the department of cardiology, and then the doctor suggested going to the respiratory department, (the doctor) let to do this (PSG). This inspection is trouble. My son-in-law did a lot for me, online registration, online payment. Maybe we (I and my husband) wouldn’t last a day on our own. But my son-in-law has to ask for leave, because we only have one baby
10	12 years	3 months	4 days	I used to snore not so seriously, then felt the brain hypoxia. I would slowly rub the back of the head, shaking the head, and slowly it would be relieved (smiling). Later, the tonsil was inflamed. After that, it (the tonsil) became larger again. In fact, it was quite serious. I went to my local hospital. The doctor said I had an enlarged tonsil and needed an operation. My daughter said she couldn’t trust him (the doctor) and wanted to come here and take a look. We both came straight away. It was only two days ago that the doctor asked me whether my snoring was serious. My daughter looked it up online and went straight to the ENT, took a scan, said the roof of my mouth collapsed, and asked me if I snore. Then I went to the respiratory department. I would have done it (PSG) last week if I had gotten my teeth fixed. My home is Panjin, far away, and I felt it troublesome
12	13 years	2 weeks	1 week	I don’t think it’s a disease. They say snoring is a blessing, a good sleep. But now, after drinking wine, there is a little suffocating feeling (my wife said). In fact, I did not want to see a doctor this time. The only problem is that I felt very tired after waking up. It is normal to feel very energetic when waking up in the morning. As for me, I felt very tired. This examination is not troublesome. My wife also said it was a disease. She is a clinician. She has been suggesting me to see a doctor, but I didn’t take seriously. To tell the truth, I have to stop to take two breaths right now. I felt there was not enough air. I don’t usually get a physical, and she (my wife) always asks me to do it. To be honest, I’m really busy, and I don’t think it’s a problem at my age
2	15 years	1 year	6 months	I accepted a small, minimally invasive procedure in the hospital of traditional Chinese medicine in 2013, but it didn’t work out well. Then I was busy and got married. I didn’t have time. This time, the main thing is that I hold my breath very badly. The snoring was so loud, you could hear it upstairs and downstairs. I’ve been gaining weight. I guessed the weight gain had something to do with it. Furthermore, I thought it was a hassle to do this test because I have been to five departments. First, ENT department. Finally, the respiratory department. I knew that snoring is a kind of disease, and I have also done minimally invasive procedures. I was mainly too busy and also did not find the right place
11	16 years	2 weeks	5 days	I’ve had two heart stent operations. This time the doctor said, “The heart is fine, maybe it is the respiratory department.” My snoring was not obvious, but there were some pauses, and I was always choking up. It is usually choked up after twelve o'clock. It seems like a nightmare, and I couldn’t move my body, which makes me uncomfortable. At this moment, I would get well and then wake up if whoever touched me. The brain also seems to be awake. When she (my wife) was not at home, I was alone, and I was afraid of the situation. I just felt that oxygen was not enough and had to wait for myself to slow down for a while. Blood oxygen was fine. I have played 120 (emergency telephone) in such a case. I had to do some tests. Furthermore, I have never taken the PSG examination before
13	20 years	3 weeks	2 years	Then, in our local hospital, a doctor suggested me to have a PSG. Because my husband was not at home, you see, there was no companion, so the booking register didn’t accept me. I have a headache every day, which is very painful. No pain is stronger than a headache. The pain almost killed me, with some vomiting and hitting the wall. Every year, I would have taken a CT and a lot of medicine, including traditional Chinese medicine. I hadn’t thought of this disease and used to think snoring was a blessing. This time the doctor said I should do a Nuclear Magnetic Resonance Imaging (MRI) and then a laryngoscope. My home is Inner Mongolia, five or six hours by train. I don’t have a companion. The doctor would dare not give me the test (PSG), so I was hospitalized
14	21 years	3 months	2 months	I’ve heard of it (OSA). I thought it didn’t matter. Furthermore, I didn’t want to come to the hospital at all. There was no cure. My wife said, “Some middle-aged men were dead because of it.” That’s why she pushed me into the hospital. I was once diagnosed with high blood pressure, so we first registered with the cardiology department. The doctor here suggested that we do this examination (PSG). I don’t think it’s too much trouble to do this test, but I think in China, with fewer medical resources and more patients
5	25 years	1 week	15 days	I also used to snore, but it’s gotten worse over the years. The voice was very loud. To tell the truth, my husband found that the apnea time was getting longer and longer. Our Yingkou hospital didn’t have such a machine and just suggested the operation. My son and daughter-in-law said, “Mother, don’t delay, go directly to Shenyang”. That’s the thing, I came over. I came to the doctor in such a situation, and the doctor said to do a test (PSG). The disease can cause a lot of cardiovascular diseases. I’m afraid of this anyway, mostly disease. I have looked into it (OSA) before. It affects life. Life is so beautiful, you know, I want to live well
6	25 years	4 days	2 weeks	I’ve been snoring for years, but it’s not serious. I didn’t know it was a disease at the time. But in the last year or two, I thought it was getting heavier and heavier with the opening mouth breathing in sleep. I wondered if there was something wrong with my throat. So, I have registered the department of otolaryngology. The doctor transferred me to the respiratory department. Then, right now, I am doing this examination. I think I am too fat, the fatter I am, the more I snore
7	50 years	3 years	3 days	I know snoring is a problem, but it’s nothing, it’s just too many illnesses, not a major one. It’s not at the top of the list. Sleep apnea disorder-what the hell, yeah, yeah, oops, it’s too long, I can’t remember it. This time because of chest tightness. I felt that chest tightness over time. My mother is an ophthalmologist, at least I also grew up in the hospital (laughing). But my father was diagnosed with heart disease. He also had sleep monitoring for snoring. I just took the test (PSG) by the way. I don’t think it is troublesome to do this test

### The intervals

The longest appraisal interval from first onset symptom to the awareness to seek medical attention is 50 years and the shortest, six months. Sometimes it is difficult for subjects to accurately quantify the appraisal interval. The help-seeking interval is generally shorter than the appraisal interval, ranging from four days to three years. The longest diagnostic interval is two years due to lack of necessary doctors and equipment or absence of a companion. The respondent 13 who had been suffering from headache delayed for 2 years, one reason was that the local hospital did not have relevant equipment, so she had to go to a superior hospital, and the other reason was that it must been accompanied to do polysomnography (PSG) in the superior hospital. The absence of the pre-treatment interval here is due to the fact that only three subjects showed a preference of treatment, which was also confirmed at the follow-ups, two respondents underwent surgery of ear, nose, and throat (ENT) department within six months and one continuous positive airway pressure (CPAP) within one year.

### Explanatory factors related to OSA

All participants in the interviews reported clinical symptoms. Two respondents (respondents 3, 10) reported suffering from throat symptoms (polyp of vocal cord, hypertrophy of tonsil), but no apnea, which wasn’t the same as five respondents (respondents 2, 5, 6, 8, 11). However, the other five respondents (respondents 4, 7, 9, 14, 15) didn’t seek medical attention until they developed various complications (gastroesophageal reflux, high blood pressure, heart trouble). The symptom of two patients interviewed (respondents 1, 12) was fatigue. Respondent 13 had been plagued by headache for years. The clinical manifestations of these non-acute symptoms affect patients’ seeking medical attention behavior to some extent.

### Factors related to patients

Most people hadn’t been conscious of the fact that they were snoring before informed by their partner or roommate. For example, respondent 4 hadn’t been aware of his snoring until his roommate told him. In addition to this, some subjects also reported periods of fatigues that were mistaken for being too tired at work. We observed two extremes of reported truths from the respondents in the interviews. One was that the patients with stronger social support had relatively shorter intervals, whose medical seeking behaviors were produced from respondents’ spouse (husband or wife) or relatives and friends. The other fact was the respondents with a family history of snoring had longer intervals. They mistook that they should be fine because the older generation in their family have been snoring for years and still be fine. Besides, the respondents with a family history were universally ​less confident in treatment.

### Factors related to health system organization

The 15 respondents were from primary hospitals, municipal hospitals and provincial hospitals according to the location of seeking medical attention. The primary hospital refers to township hospitals, village clinics and community hospitals that provide basic public health services and basic medical services, mainly serving rural residents. There are few outpatient clinics for OSA in primary hospitals. We found that there was no respondent had been referred by primary hospitals except for respondent 5. Most people were referred from municipal hospitals, which are mainly managed by the municipal-level health system organization and provide medical and health services to residents in the entire municipal area. Some municipal hospitals in part don’t have enough the diagnostic or therapeutic equipment. Thus, they tended to prefer provincial hospitals encouraged by municipal hospitals. But, for provincial hospitals, there were fewer beds per capita in the sleep laboratory room or center available for appointment more patients. The main reason is that provincial hospitals provide health services within the province and throughout the country, which are usually directly managed by the provincial-level health system organization. These factors prolonged the patient’s diagnosis delay to a certain extent.

## Discussion

### Strengths and limitations of the study

The study was based on the theory of the Model of Pathways to Treatment. The application of the Model of Pathways to Treatment in the study made up for the lack of theoretical support in previous studies on the delay in medical treatment of patients with OSA. According to the theory, the patient’s delay was divided into appraisal interval, help-seeking interval, diagnostic interval. And most importantly, we were able to clearly understand the most obvious part of the patient’s delay, which was the appraisal interval. After that, we have analyzed the factors that affected the delays in seeking medical attention from the perspectives of the individual, the disease, and the health service system. So, the results provided a powerful intervention direction for future research and the public health administration. However, one thing to be clear was that the self-reported intervals in the interviews were estimated by the events of special significance in their lives, which was impossible to pinpoint the exact date.

### Time elapsed before diagnosed and determining factors

It can be seen that the delay rate for patients with OSA in seeking health care is relatively high. The interviews show that the shortest delay interval for patients with OSA to seek health care behavior is 0.5 years, all of which are more than 3 months, with a delay rate of 100% and the longest time being 50 years. As far as the respondents were concerned, they often didn’t aware the fact that they were snoring during the sleep, the leading cause of delayed medical treatment. They didn’t realize it until they were told by their spouse or roommate. Furthermore, traditional wisdom holds that snoring is a sign of good health and happiness, or the snoring is a blessing in Chinese culture. In the situation, they don’t know what OSA is and how harmful it is to the target organ. They assumed that snoring was not a big deal even though they already knew that they were snoring in the sleep, which was most obvious in the 13 patients with a family history of snoring. It’s not surprising that the subjects with the false perception had a long delay in seeking medical advice. What’s more, the subjects with lower social support, were more likely to delay in seeking medical treatment than those with higher social support mainly from their spouses [5, 9–11]. Besides, the subjects often use busyness as an excuse to delay seeking medical attention [12]. However, this phenomenon is more evident in the middle-aged and elderly than in the young and middle-aged. Patients in the young and middle-aged, the life stage of “having both parents and young children to support and take care of”, may pay more attention to the health because a strong sense of responsibility forces them to keep healthy, which promotes them to seek medical treatment as soon as possible [13]. Factors related to the disease itself are as follows: firstly, our results showed that atypical or vague symptoms of OSA led to longer pre-diagnostic intervals, except for those typical symptoms, such as frequent suffocation in the sleep or falling asleep while waiting for a traffic light. The symptoms are usually little by little aggravated, not as urgent as a heart attack or a brain hemorrhage, which are usually life-threatening. The respondents reported that their symptoms were daytime sleepiness but far from affecting their daily lives [14]. None of the respondents had ever been to the emergency room for their symptoms. Furthermore, it’s well known that OSA and obesity are mutually causal. Most of the obese patients thought their symptoms would ease after losing weight. The health system’s organization mainly affect the delay in diagnostic interval and pre-treatment interval of patients with OSA. The study indicated that health resources were unequal: some patients complained that local hospitals had a lower medical level and were far away from superior hospitals, for that reason, there were few integrated prevention and control networks of hospitals at all levels. Some factors resulting in patients not receiving timely diagnosis and treatment have been shown, such as imperfect sleep monitoring equipment, a lack of multidisciplinary sleep diagnosis centers, fewer sleep monitoring beds per capita, and complex medical procedures in hospitals with inconvenient emergency room green paths, a longer waiting time for examination, and insufficient understanding of the OSA for some medical staff [15–18]. The medical treatment behavior of OSA patients would have been affected by country medical insurance policies, reimbursement modes and proportions of provinces and cities, and coverage of private medical insurance [18–23]. For example, the government fully bears the treatment cost of OSA in France, while it only partially reimburses the surgical treatment cost of OSA in China. Its first-line treatment, CPAP, is still self-funded in China. Now, Polysomnographic monitoring is not included in routine hospital physical examinations or in special populations, for example, drivers and obese people. Thus, it is recommended that PSG should been included in routine physical examination for patients with obesity or other special conditions, even if they have no symptoms.

### Avenues for improving early OSA diagnosis

National health authorities should make corresponding publicity for patients, so that the general public gradually understand the OSA and its harm, and the corresponding treatment. Medical-seeking behavior is primarily the result of personal knowledge, attitude/belief and practice [24, 25]. Compared with chronic diseases such as hypertension and diabetes, OSA has a lower awareness rate [26]. The results indicated that eight participants (respondents 3, 6, 9, 10, 11, 12, 13, 15) were unaware of OSA, six participants (respondents 4, 7, 8, 1, 2, 14) had heard of OSA as a disorder but did not take it seriously, and only one participant (respondents 5) had known about it. One in 15 participants opted for CPAP within one year, which is far lower than the 50 percent reported [27]. The damages and complications of OSA to target organs cannot be recognized properly and in time [28], and then timely treatment is not possible after diagnosis. Patients may choose treatment only when accompanied by severe symptoms, resulting in a lower treatment level. At the same time, the medical staff’s cognition of OSA should also be improved to ensure the alertness of OSA [29, 30]. Our analysis indicated that it is possible to prompt early medical treatment and standardize treatment management for patients with OSA, provided that awareness of OSA is increased.

Diseases have their own natural evolutionary progression, which no intervention can change. In this case, we can’t change the path of it, but can only detect it early. Currently, Polysomnography is not included in routine medical examinations in China. Thus, we recommend that different strategies are needed for the different types of people in routine medical check-ups. For special high-risk occupational groups, like a driver, OSA screening for every commercial driver is advocated, given under-resources medical system. In addition, Polysomnography test should be prescribed by clinicians in routine medical check-ups for those special populations who have a short neck with small jaws, rhinitis, excess fat with a neck circumference of more than 40 cm [31].

In addition, the state and the government should vigorously adjust medical resources and strengthen the attention to primary hospitals by improving the relevant diagnostic equipment to improve the situation. It is necessary to establish a diagnosis and treatment system suitable for grassroots work and train medical teams that can stick to working at the grassroots level for a long time and are familiar with the diagnosis and treatment technology of OSA. It is more important to establish a comprehensive prevention and control network based on grassroots medical institutions and coordinated by hospitals at all levels. And further national health authorities need to increase the reimbursement rate for the treatment of OSA, both surgical and CPAP for further improving situation of OSA patients’ medical treatment. Let it be sooner rather than later.

## Conclusions

This study illuminated the influencing factors of the elapsed time between first symptom and diagnosis to be longer for some patients than others. These influencing factors can be broadly divided into three categories, namely, disease characteristic, patients, and health service system organization. The OSA does not cause serious complications in a short time, and only affects life when it progresses to a serious degree and the special characteristics of it have led to a general lack of attention to OSA. Besides, patients have serious cognitive deficits and deep-rooted beliefs about OSA in Chinese culture. And one more important thing, there are significant inequalities in access to health resources and services. We hope that the results can provide a reference for national and local medical policies and solutions.

## Data Availability

The datasets analyzed during the current study are not publicly available due to the fact that it might create a breach in participant confidentiality but are available from the corresponding author on reasonable request.

## Abbreviations

OSA: Obstructive sleep apnea
AHI: Apnea hypopnea index
HCP: Healthcare professional
LSpO_2_: Lowest saturation of peripheral oxygen
PSG: Polysomnography
ENT: Ear, nose, and throat
CPAP: Continuous positive airway pressure
MRI: Nuclear magnetic resonance imaging
